# Human anthrax in a non-epizootic area: epidemiological investigation and response based on One Health—Chengdu, China

**DOI:** 10.3389/fpubh.2025.1683530

**Published:** 2025-10-30

**Authors:** Kai Xu, Yao Wang, Shihong Liu, Jiang Han, Rong Zhou, Wenjia Tian, Yang Yang, Liang Wang

**Affiliations:** ^1^Chengdu Center for Disease Control and Prevention, Chengdu, China; ^2^Pengzhou Center for Disease Control and Prevention, Pengzhou, Chengdu, China; ^3^Pidu Center for Disease Control and Prevention, Pidu, Chengdu, China

**Keywords:** anthrax, non-epizootic, One Health, outbreak investigation, risk control

## Abstract

**Background:**

Anthrax, caused by *Bacillus anthracis*, is endemic in western China’s pastoral regions. Urban areas adjacent to these regions face a growing threat from the unregulated or poorly monitored livestock trade. This study reports the first documented outbreak of cutaneous anthrax in Chengdu, a non-epizootic city, which originated from the slaughter of infected cattle imported from an epizootic area.

**Methods:**

A multidisciplinary team applied the “One Health” approach to investigate the outbreak. The investigation included case details, symptoms, laboratory results, potential sources of infection, suspected contaminated environments, local natural landscapes related to animal husbandry practices, disease incidence rates, slaughtering process, and vaccination history. A risk assessment focused on human, animal, and environmental factors to guide containment measures.

**Results:**

Two cutaneous anthrax cases were confirmed, epidemiologically linked to the unprotected handling of cattle imported from Aba Prefecture. *B. anthracis* was detected via qPCR in samples from a patient’s skin lesions, beef, viscera, and forage; environmental samples were negative. Blood cultures showed no bacterial growth. Interventions included disinfection (10,000 mg/L chlorine), livestock culling, and incineration of traced beef. Approximately 30% of sold meat remained untraceable due to cash transactions, indicating surveillance gaps. Initial misdiagnosis as “insect bites” delayed confirmation by 4–6 days. Both patients recovered following antibiotic treatment, developing eschars with no fatal outcomes. Environmental assessments indicated ongoing risk due to unsealed soil and poor biosecurity.

**Conclusion:**

This outbreak underscores the systemic risk of anthrax in non-epizootic urban areas due to unregulated or poorly monitored livestock trade and poor farm biosecurity. While the One Health approach enabled effective containment, it revealed critical gaps in market oversight and diagnostics. Key recommendations include implementing integrated surveillance, mandatory electronic tracing, training for healthcare workers, and stricter quarantine enforcement to prevent zoonotic spillover.

## 1 Introduction

Anthrax is a zoonotic disease caused by *Bacillus anthracis*, a spore-forming bacterium characterized by its environmental stability and ubiquitous distribution in nature (1). Herbivores are the primary natural hosts. Humans acquire the disease incidentally through contact with infected animals or their products. Cutaneous anthrax is the most prevalent form, accounting for over 95% of human cases (2). This form typically manifests as a localized skin infection, most frequently occurring on exposed areas such as the face, neck, arms, and hands. The characteristic skin lesion begins as a pruritic papule, which evolves into a vesicle and ultimately forms the classic black, necrotic eschar (3).

In China, high-risk regions for anthrax are predominantly located in the western part of the country, such as Qinghai, Sichuan, Ningxia, Xizang (Tibet), and Xinjiang (4, 5). These areas are characterized by extensive grazing and breeding of cloven-hoofed animals, primarily cattle and sheep. Consequently, anthrax cases primarily affect young male herdsmen involved in agriculture and animal husbandry. These cases predominantly present as the cutaneous form, which is characterized by high incidence but low mortality (6). These epidemiological features often lead governments and public health agencies to focus intervention efforts on high-risk populations in endemic rural areas, while overlooking urban areas with high levels of beef and mutton consumption. Such urban areas typically have substantial populations engaged in livestock slaughtering, processing, and related occupations, in addition to hosting numerous small-scale animal breeding operations.

Although documented cases of anthrax in non-epizootic urban areas are scarce, high consumption of beef and mutton, coupled with potential lapses in meat safety regulation, poses a persistent threat of anthrax introduction, particularly in urban areas adjacent to pastoral regions. This report describes sporadic cases of cutaneous anthrax identified in Chengdu, Sichuan Province, originating from the slaughter of cattle that had succumbed to anthrax. Sichuan Province consistently reports one of the highest incidence rates of anthrax nationally. Major hotspots include the Ganzi, Aba, and Liangshan autonomous prefectures, where pastoralism, predominantly managed by ethnic minority communities, supplies the majority of beef and mutton to Chengdu. In recent years, cutaneous anthrax has been the predominant form involved in clustered outbreaks within Sichuan, with transmission primarily linked to the slaughtering, handling, or consumption of infected animals. This study highlights the neglected intersection between human public health and animal health systems, reveals limited awareness among livestock workers in non-epizootic areas, and underscores the critical importance of the One Health framework and collaborative cross-sectoral mechanisms for effective anthrax prevention and control.

## 2 Methods

### 2.1 Case definitions

#### 2.1.1 Suspected case

A case meeting at least one of the following criteria:

(a) Presence of typical cutaneous anthrax lesions.
(b) Other clinical manifestations suggestive of anthrax, along with a relevant epidemiological exposure history.

#### 2.1.2 Clinically diagnosed case

A suspected case meeting at least one of the following criteria:

(a) Microscopic examination reveals Gram-positive rods with truncated ends appearing in a chain-like arrangement.
(b) Positive detection of *B. anthracis* antigen in body fluid or secretion specimens.
(c) Positive detection of antibodies against *B. anthracis* in blood specimens.
(d) Exposure to animals diagnosed with anthrax or culture of *B. anthracis* from environmental exposure specimens.

#### 2.1.3 Confirmed case

A suspected or clinically diagnosed case meeting at least one of the following criteria:

(a) Isolation and identification of *B. anthracis* by culture.
(b) Detection of *B. anthracis* specific nucleic acid by polymerase chain reaction (PCR).
(c) Seroconversion of specific antibodies against *B. anthracis* toxins or a ≥ 4-fold rise in antibody titer between acute- and convalescent-phase serum samples.
(d) Meeting at least two of the following criteria: Microscopic examination reveals Gram-positive rods with truncated ends appearing in a chain-like arrangement; Positive detection of *B. anthracis* antigen; Positive detection of *B. anthracis* antibodies; Isolation of *B. anthracis* from exposed animal or contaminated environmental sample.

### 2.2 Data collection

Data were collected from multiple sources, including field epidemiological investigations, interviews (with the patients, local villagers, and healthcare personnel), and reviews of medical records and laboratory reports from the Center for Disease Control (CDC). The collected data encompassed case demographics, timeline of onset and treatment, clinical manifestations, potential contact and exposure history, environmental contamination assessment, identification of at-risk personnel, and details on animal sourcing, slaughter procedures, morbidity/mortality, and carcass management. Additionally, vaccination records were retrieved and reviewed.

### 2.3 Outbreak investigation and team structure

A multidisciplinary rapid response team (RRT) was established, comprising experts from public health, veterinary medicine, forestry, and other relevant sectors. Guided by the One Health framework, the team conducted the outbreak investigation by collecting the comprehensive data outlined in section 2.2. A risk assessment was subsequently performed, focusing on three critical dimensions: human cases, animal sources of infection, and environmental factors. The findings from this assessment informed the development of outbreak management strategies and risk control measures (Table 1).

**Table 1:** Division of responsibilities among joint prevention and control task force departments.

Member department	Responsibilities
Public health and clinical medicine	1. Case identification, isolation, and treatment
	2. Epidemiological investigation
	3. Sample collection and laboratory testing
	4. Terminal disinfection of contaminated environments
	5. Health education and emergency monitoring
	6. Epidemic data compilation and reporting
Animal disease control	1. Harmless disposal of infected livestock
	2. Comprehensive disinfection of farms
	3. Collection and cross-regional reporting of animal epidemic information
Market supervision and local administration	1. Tracking livestock sources and distribution (verification of quarantine certificates, investigation of transaction records)
	2. Market recall and destruction of contaminated products (beef, offal)
	3. Promotion and enforcement of policies and regulations
	4. Registration and routine supervision of small-scale farms
Public security	1. Technical support (surveillance retrieval, electronic tracking of contaminated beef distribution)
	2. Personnel deployment and order maintenance
	3. Assistance in investigating illegal transactions (unquarantined livestock circulation)
Forestry and natural resources	1. Environmental monitoring of epidemic foci
	2. Assessment of spore contamination in soil and water sources
	3. Risk investigation of anthrax in wildlife

The specific responsibilities assigned to each sector within the RRT were as follows:

Public health & clinical medicine: Case identification, isolation, diagnosis, and treatment; epidemiological investigation and contact tracing; specimen collection and surveillance; environmental disinfection; health education; and emergency monitoring.
Animal disease control: Safe disposal of infected livestock (carcasses); disinfection of affected farms; and reporting of animal epidemic information.
Market supervision & local administration: Tracing the origin and movement of livestock; disseminating and enforcing relevant policies and regulations.
Public security: Provision of technical and personnel support.
Forestry & natural resources: Environmental monitoring of epidemic foci; assessment of *B. anthracis* spore contamination in soil and water sources; and investigation of anthrax risks in wildlife.

In addition to the multidisciplinary team, local community administrators and resident neighbors were invited to participate in semi-structured interviews. This engagement with the community enabled a rapid situational assessment and facilitated the formulation and implementation of targeted response measures. Furthermore, the case patients were asked to visually identify key exposure locations, including the slaughter site, via video conference.

### 2.4 Laboratory testing

A range of specimens were collected from human cases, animals, and the environment for laboratory analysis to confirm the identity of the pathogen and sources of contamination.

#### 2.4.1 Human samples

Venous blood samples (5 mL per case; *n* = 2) were collected aseptically from each patient. Each sample was divided into two aliquots: one for standard bacteriological culture and the other for nucleic acid detection via quantitative real-time PCR (qPCR). Sterile swabs moistened with physiological saline were used to obtain samples from the skin lesions of each patient (two swabs per case; total of two cases). These swab samples were subjected to nucleic acid testing.

#### 2.4.2 Animal and environmental samples

The following samples were collected for nucleic acid detection: visceral organs from the deceased bovine, meat from the same animal, surface soil from the cattle farm, blood-stained cloth, forage, and wastewater from the periphery of the cattle pen.

Nucleic acids were extracted from all specimens using an SSNP-3000A automated extraction system with the ShuoShi Bacterial DNA Extraction Kit (Magnetic Bead Method), with all procedures conducted within a HFsafe-1200L CB2 biosafety cabinet.

Subsequent quantitative real-time PCR (qPCR) was carried out on a Roche Light Cycler 480II instrument using a commercial duplex assay kit (Zhuocheng Huisheng) to simultaneously detect two *B. anthracis*-specific virulence genes: the protective antigen gene (pagA, FAM channel) and the capsule gene (cap, VIC channel). The thermal cycling protocol was as follows: initial denaturation at 95 °C for 30 s, followed by 40 cycles of 95 °C for 5 s and 60 °C for 30 s, with fluorescence signal acquisition at the 60 °C step.

For result interpretation, a sample was considered positive if… with a cycle threshold (Ct) value of ≤35. Samples yielding Ct values between 35 and 40 were deemed indeterminate and subjected to repeat testing; a reproducible amplification with Ct ≤40 upon re-testing was confirmed as positive.

## 3 Results

### 3.1 Case descriptions

Two male patients were identified in this outbreak. Their clinical profiles are summarized below:

#### 3.1.1 Case 1 (confirmed case)

A 37-year-old male, a long-term resident of Aba Prefecture who co-managed a small-scale cattle farm in Chengdu. On September 26, 2024, he developed a erythematous, indurated papule on his right forearm, initially resembling an insect bite. On September 27, he developed a fever with a recorded rectal temperature of 39.0 °C. By September 28, the lesion had evolved into five vesicles on the right forearm, surrounded by erythema. Concurrently, swelling, pain, and localized warmth extended proximally from the forearm to the mid-upper arm. Several vesicles ruptured, discharging serous fluid. He initially presented at a local clinic on September 26, where the lesion was misdiagnosed as a “mosquito bite.” He was treated symptomatically with cephalosporin and ibuprofen. However, upon presentation to a different hospital on September 30, a diagnosis of “suspected cutaneous anthrax” was made, leading to his immediate hospitalization for isolation and targeted treatment. Following admission, his condition improved, with stabilization of vital signs.

#### 3.1.2 Case 2 (clinically diagnosed case)

**Figure 1:**
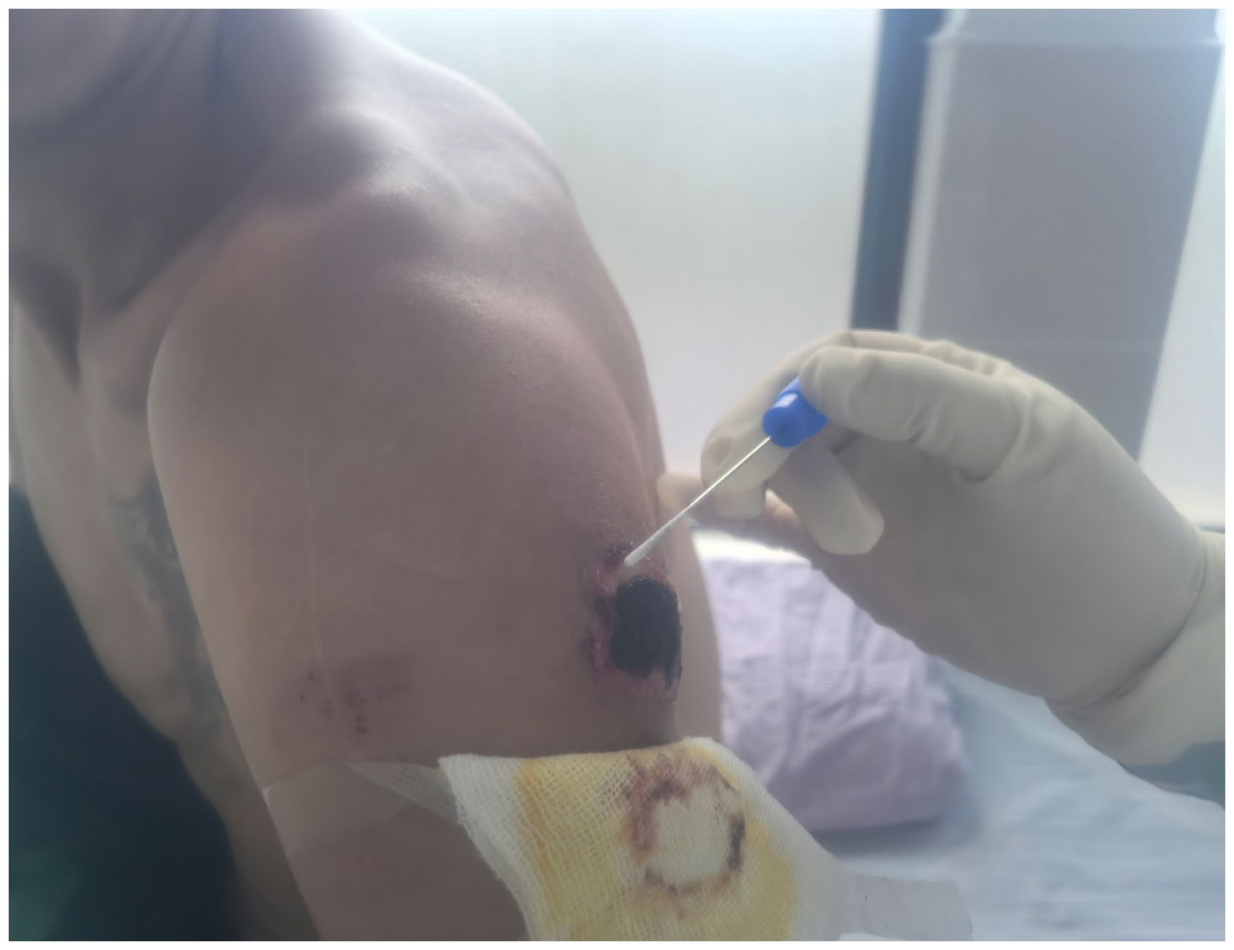
Black scab on the outside of the left upper arm in case 2.

A 35-year-old male employed at the same farm. On September 24, he noted discomfort on his left upper arm while working at the cattle farm. This was followed by the gradual onset of pain, localized erythema, pruritus, and ulceration. The patient attempted self-treatment by incising the lesion with a knife, resulting in exacerbated pain and swelling, accompanied by left axillary lymphadenopathy (swollen and tender lymph nodes). On September 27, he developed a fever (axillary temperature 38.0 °C) and generalized fatigue, prompting hospital admission. Physical examination and laboratory findings upon admission included: leukocytosis (white blood cell count 9.46 × 10^9^/L) with neutrophilia (69.4%), elevated random blood glucose (9.4 mmol/L), trace urine protein, and confirmation of enlarged left axillary lymph nodes by color Doppler ultrasound. He was initially diagnosed with “skin infection and axillary lymphadenitis” and received intravenous metronidazole and piperacillin sodium. His symptoms subsequently improved, and the skin lesion progressed to form a characteristic black eschar (Figure 1).

Neither patient had a history of prior anthrax infection or vaccination.

### 3.2 Epidemiological findings

**Figure 2:**
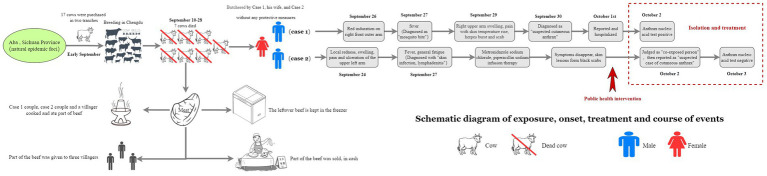
Schematic diagram of exposure, onset, treatment and course of events.

In early September 2024, Case 1 purchased a total of 17 cattle in two separate batches from Hongyuan County, Aba Prefecture, and transported them to a farm in Chengdu for breeding. The animals were acquired without valid official quarantine certificates. Beginning on September 10, seven of these cattle died successively. The carcasses were subsequently butchered on-site at the farm by Case 1, his wife, and Case 2. These high-risk procedures were performed in the absence of any personal protective equipment (PPE). During this process, Case 1 sustained a hand injury (Figure 2).

### 3.3 Environmental assessment and disinfection measures

The farm consisted of a semi-open shed located in an open area, with only one neighboring household in proximity. The floor of the structure was partially cemented and partially earthen. In addition to the animal pens, the facility was used for storage of various materials, including hay piles, animal feed, water buckets, and nylon bags.

The on-site butchering of all seven cattle resulted in extensive environmental contamination, characterized by:

(1) Blood seepage into the underlying soil and drainage channels.
(2) Splash contamination of surrounding haystacks, nylon bags, and fencing.

These contaminated materials, along with other organic matter present at the site, represent potential substrates for long-term pathogen persistence and spore maturation.

Terminal disinfection was performed by spraying the entire farm environment with a disinfectant solution containing 10,000 mg/L available chlorine. Treated areas included the cattle stalls, floors, and all other identified contaminated sites. Heavily contaminated materials, such as hay and nylon fabrics, were collected and incinerated. Additionally, clothing belonging to the cases was sterilized by autoclaving.

### 3.4 Beef traceability and distribution

Following dissection, meat from the seven infected cattle was processed on-site. A portion of this meat was cooked and consumed by the two cases, their household members, and one other villager. Additionally, quantities of beef and offal were distributed to three additional villagers. An estimated minimum of 50 kg of beef (the exact quantity was unrecalled) was sold via cash transactions to unidentified buyers. The remainder was stored in a freezer.

The absence of formal records and reliance on cash transactions significantly hampered comprehensive traceability efforts. Through coordinated efforts with the public security, market regulation, and relevant industry departments, a portion of the contaminated beef was successfully traced and retrieved. All recovered meat was subsequently incinerated. However, a portion of the beef sold via cash transactions could not be traced or recovered.

The remaining live cattle on the farm were safely disposed of following the establishment of compensation agreements, thereby eliminating any further risk of transmission.

### 3.5 Laboratory results

#### 3.5.1 qPCR detection

Skin lesion swabs from both cases and samples of contaminated beef and forage tested positive for *B. anthracis*. The cycle threshold (Ct) values for the cases’ swabs were as follows: Case 1 (34.23, 33.91) and Case 2 (37.16, 37.42). All other environmental and clinical samples returned negative results.

#### 3.5.2 Bacterial culture

Blood cultures from both patients yielded no growth of *B. anthracis*, consistent with cutaneous anthrax pathophysiology.

### 3.6 Contact tracing and public health response

Three individuals, all co-residents of the index cases, were identified as close contacts. An additional five individuals were classified as general contacts due to either indirect exposure to contaminated raw beef or casual contact with a confirmed case. With the exception of the two confirmed cases, none of the identified contacts developed any relevant clinical symptoms during the observation period. Support was provided to the animal health authorities for the trace-back investigation of the outbreak to its source in Aba Prefecture. Comprehensive health education and a 14-day medical observation period were mandated for all individuals with a history of exposure, encompassing both close and general contacts.

It has been recommended to health care institutions at all levels to enhance surveillance and clinical vigilance against anthrax. Clinicians should remain highly suspicious of patients with compatible skin lesions such as papules, vesicles or eschar, and regularly inquire about potential epidemiological exposure risks, including contact with livestock or animal products.

## 4 Discussion

Based on a comprehensive analysis of laboratory findings, epidemiological investigations, and environmental assessments, we conclude that this event represents a sporadic human case of cutaneous anthrax. The infection originated from the slaughter of cattle that had died of anthrax, which were imported from an endemic area. These are the first reported human anthrax cases in Chengdu, demonstrating a clear trajectory of risk transmission from animal anthrax-endemic regions to a non-epizootic urban area. Several factors contributed to this outbreak. Primarily, the absence of valid quarantine certificates for the involved cattle underscores potential regulatory loopholes in the management of live animal trading markets. Indeed, similar anthrax outbreaks linked to the purchase, slaughter, and processing of unquarantined livestock—sourced from both formal markets and private transactions—are frequently documented (7–9). Furthermore, a critical lack of knowledge and awareness regarding anthrax prevention and control among livestock workers significantly elevated the risk of zoonotic transmission. This is consistent with established literature indicating that most human anthrax cases result from direct contact with infected animals or their tissues during activities such as slaughtering, butchering, and skinning (10). In this study, the patients performed the dissection of the cattle without employing any personal protective measures, despite harboring suspicions that the animals were diseased.

### 4.1 Cross-regional transmission and urban risks of anthrax

This outbreak was traced to the purchase of unquarantined cattle from Aba Prefecture, a recognized anthrax-endemic area. This event underscores the persistent risk of cross-regional transmission resulting from insufficient oversight of the live animal trade between epizootic and non-epizootic zones. Similar incidents involving unregulated animal trade and a lack of protective measures have been documented in other Chinese provinces, including Guizhou (6), Shandong (8), and Liaoning (10). As a major livestock-producing region in Sichuan, Aba Prefecture can act as an epidemiological bridge, facilitating the dissemination of pathogens when animals are transported to consumer cities like Chengdu in the absence of rigorous quarantine certification and traceability mechanisms.

The emergence of anthrax cases in a major metropolitan and historically non-epizootic area like Chengdu signals a distinct and emerging public health threat. Urban environments present unique risks for outbreak amplification due to high population density, complex food supply chains, and the inherent challenges in tracing contaminated products—particularly those sold through informal, cash-based transactions. Furthermore, small-scale urban and peri-urban farms often lack adequate infrastructure, as exemplified by the unpaved ground in this case, which compounds the risk of environmental contamination and exposure. Soil contaminated with blood or other infectious tissues can facilitate long-term spore persistence, creating a reservoir for secondary transmission even if initial environmental samples test negative.

This investigation demonstrates that mitigating outbreak risk in urban settings requires a pre-planned and agile response strategy. Moving forward, consumer cities like Chengdu should develop and implement standardized response protocols specifically designed for zoonotic diseases introduced via imported livestock. Strengthening surveillance at critical nodes along the livestock supply chain, rigorously enforcing existing regulations, and adopting integrated One Health approaches are imperative for preventing future outbreaks.

### 4.2 Lack of knowledge and protective behavior among employees

Both Case 1 and Case 2 participated in dissecting a deceased bovine without employing any personal protective equipment (PPE). They handled the carcass with bare hands, despite the presence of pre-existing hand wounds in Case 1, which directly led to their cutaneous anthrax infections through percutaneous inoculation. This high-risk behavior underscores a critical knowledge gap regarding anthrax transmission and prevention among livestock workers. This finding is consistent with studies reporting that livestock workers generally possess limited knowledge of anthrax, including its clinical signs in animals, transmission routes, and essential preventive measures. For instance, a study in West Kazakhstan found that while 91% of respondents were engaged in agriculture and 67% raised cattle, over 50% were unaware of the zoonotic potential of anthrax. Moreover, approximately 82 and 87% could not recognize its clinical signs in animals and humans, respectively (11). A similar deficiency in anthrax knowledge has been documented among livestock workers in China. For example, during an outbreak in Jiangsu, none of the seven affected individuals used protective measures during slaughtering activities (12). Similarly, a case report from Shandong described a patient who purchased, slaughtered, and sold sick cattle without quarantine certificates; notably, all participants involved in the slaughter process operated without any protective measures (9). These cases collectively highlight a critical disconnect between knowledge and practice, often described as the “knowing-doing gap.” Therefore, implementing regular, targeted training for livestock workers in non-epizootic areas is crucial. Such training must emphasize core principles, including the immediate reporting of suspected animal cases and the standardized disposal of carcasses, to effectively block the transmission pathways of zoonotic diseases like anthrax.

### 4.3 Diagnostic challenges for cutaneous anthrax in primary care settings

Case 1 was initially misdiagnosed with an “insect bite” at a local clinic, resulting in a critical delay in appropriate treatment and public health reporting. This misdiagnosis underscores a limited familiarity with the clinical progression of cutaneous anthrax among frontline healthcare providers. Although the disease typically evolves from a painless papule to a characteristic black eschar, these signs can be overlooked by clinicians in non-epizootic areas due to a lack of clinical exposure. Similar diagnostic delays have been documented in other non-epizootic regions of China. During an outbreak in Jiangsu province, the index case visited multiple healthcare facilities (village, township, and county levels) but was not correctly diagnosed with anthrax until 1 week after symptom onset (12). Likewise, a case report from Shanghai described a patient who sought care at different hospitals across three cities over 4 days, receiving successive diagnoses of “soft tissue disease,” “insect bite with infection,” and “limb infection” before being correctly identified as a cutaneous anthrax case on the tenth day (13). To enhance early diagnostic capacity at the primary care level, we propose the following measures: (a) Integrate anthrax into continuous medical education programs for clinicians in non-epizootic areas, emphasizing its inclusion in the differential diagnosis of ulcerative skin lesions. (b) Establish a regional expert consultation network for infectious diseases, utilizing telemedicine platforms to facilitate rapid specialist review of suspected cases from grassroots institutions. (c) Strengthen the coordinated reporting mechanism between medical facilities and the Center for Disease Control (CDC) to ensure immediate reporting and laboratory testing of all suspected anthrax cases.

### 4.4 The value of participatory epidemiology in outbreak response

This study highlights the critical role of participatory epidemiology (PE) in enhancing grassroots outbreak investigations. While conventional surveillance can outline transmission chains, it often overlooks the socioeconomic and behavioral drivers of disease spread. By involving livestock farmers, community residents, and local administrators in reconstructing exposure events and control priorities, we identified key risk pathways and collaboratively developed contextually relevant interventions. The application of PE enables more effective identification of high-risk behaviors, tracing of cryptic transmission pathways, and co-design of contextually appropriate and socially acceptable intervention strategies (14, 15). Integrating PE principles into routine training for frontline personnel in non-epizootic areas is essential for building community trust and strengthening local response capacity (16). We recommend establishing institutionalized PE mechanisms within local disease control and animal health centers to facilitate regular dialogue and risk assessment with high-risk groups. Such engagement helps translate local observations into actionable intelligence, addressing key gaps in conventional surveillance and supporting more adaptive, community-centered zoonotic control.

### 4.5 Effectiveness and challenges of the One Health mechanism

The “One Health” concept is a multi-sectoral strategy widely recognized as an effective framework for fostering collaborative, sustainable, and realistic initiatives to achieve optimal health outcomes for humans, animals, and the environment (17). The successful containment of this outbreak exemplifies the efficacy of this approach, which was centered on interrupting the transmission chain through three core components: (a) source containment through cross-sectoral collaboration to trace and remove contaminated animal products; (b) exposure management via rapid identification of exposed individuals and close contacts, supported by prophylactic treatment; and (c) environmental decontamination through terminal disinfection to eliminate persistent spores and prevent environmental reservoirs. This coordinated effort involved joint operations across public health, veterinary, agricultural, public security, and market regulation agencies, encompassing contact tracing, environmental disinfection, product tracking, and risk assessment. This successful interagency collaboration underscores the indispensable role of the One Health framework in effective zoonotic disease control.

Despite this success, the response revealed critical systemic shortcomings that highlight areas for improvement. First, regulatory gaps in the live animal trading market permitted the cross-regional movement of cattle without valid quarantine certificates. This exposes weaknesses in interdepartmental oversight and underscores the urgent need to strengthen joint enforcement mechanisms involving animal husbandry, transportation, and public security authorities. Second, the inability to trace contaminated beef sold through informal cash transactions revealed critical vulnerabilities in the food traceability system, a challenge particularly acute in non-epizootic urban areas. To address these gaps, lessons can be adopted from the European Union’s integrated “farm-to-fork” traceability model to enhance overall supply chain transparency. The promotion of electronic quarantine certificates and the exploration of blockchain technology present promising avenues for establishing real-time monitoring of livestock movement (18).

### 4.6 Strengthening anthrax preparedness and response in non-epizootic urban areas

The increasingly common anthrax epidemic in cities in non-epizootic areas has sounded the alarm for similar areas. We propose a multi-faceted strategy to optimize preparedness and control, focusing on the following key areas:

(1) Enhanced risk monitoring: Enhancing risk surveillance systems should be prioritized, with particular emphasis on establishing sentinel surveillance points at critical control points along the livestock supply chain, including trading markets, slaughterhouses, and farms. This surveillance system should incorporate regular environmental, sewage and animal sample testing for *B. anthracis* spore detection to ensure early detection of potential outbreaks.
(2) Legislative and policy support: Developing and enforcing a strong legislative framework is essential to support quarantine measures, vaccination, market regulation, and coordination between different health sectors. Integrating the “One Health” principle into health policies ensures a coordinated approach to the prevention and control of zoonotic diseases.
(3) Public health education: Popularize anthrax prevention and control knowledge for livestock practitioners, meat processors and consumers through short videos, community lectures and other forms, emphasizing the principle of “no slaughtering, no eating, no trading of dead animals.”
(4) Training of diagnosis and treatment personnel: Strengthen anthrax knowledge training for medical personnel in diagnosis and treatment institutions, especially primary medical institutions, to improve the ability of early identification of anthrax and diagnosis awareness.

### 4.7 International trade risks and advice

As a leading global importer and exporter of livestock and animal products, China requires robust and resilient biosecurity systems to mitigate the risk of cross-border transmission of anthrax and other zoonotic diseases. The vulnerabilities in domestic quarantine and traceability systems exposed by this outbreak could potentially facilitate the unintentional international movement of infected animals or contaminated goods. To mitigate these risks, China should prioritize the nationwide implementation of an integrated, digitally-enabled traceability system that tracks livestock and products from point of origin to final destination. Concurrently, it is critical to strengthen border inspection protocols and enhance laboratory capacity for the rapid detection of *B. anthracis* in both imported and exported commodities. Furthermore, proactive international collaboration is indispensable. China should actively engage in multilateral information-sharing platforms and align its sanitary and phytosanitary measures with international standards to harmonize global risk management efforts.

### 4.8 Study limitations

This study has several limitations. First, incomplete traceability of contaminated beef—primarily resulting from unrecorded cash transactions—likely resulted in undetected exposures among individuals outside the scope of our management efforts. Second, although environmental samples (e.g., soil and sewage) tested negative for *B. anthracis*, the possibility of persistent spore contamination cannot be entirely excluded. Factors such as sampling timing, spatial heterogeneity of contamination, and the detection limits of the assays may have contributed to these false-negative results. Thus, the potential for long-term spore persistence, especially in soil, remains a concern and highlights the need for continued environmental monitoring. Finally, the baseline knowledge and risk perceptions of livestock workers were not quantitatively assessed; future studies should utilize structured questionnaires to systematically evaluate cognitive gaps and underlying behavioral drivers.

## 5 Conclusion

This outbreak demonstrates that the risk of anthrax introduction into non-epizootic areas is substantial and should not be dismissed as a low-probability event. This risk is intrinsically linked to geographical proximity to endemic zones, the volume of live animal trade, and the integrity of local surveillance and control systems. The integrated One Health approach—spanning multi-sectoral coordination, professional training, legislative reinforcement, and technological innovation—proves essential for strengthening zoonotic disease control and mitigating the risk of future outbreaks.

This study reveals a critical knowledge gap concerning anthrax among livestock workers in non-epizootic regions and underscores operational vulnerabilities within small-scale farming systems. Furthermore, it validates the critical function of the One Health framework in orchestrating coordinated actions across public health, veterinary services, and market governance. Our findings indicate that anthrax surveillance and preparedness must extend beyond recognized endemic regions to include adjacent pastoral areas and high-consumption urban centers, which remain susceptible to sporadic introductions. Therefore, enhanced education and training for breeding and slaughtering personnel, along with stronger inter-sectoral cooperation, are critical priorities.

## Data Availability

The original contributions presented in the study are included in the article/supplementary material, further inquiries can be directed to the corresponding author.
